# Spatial transcriptomics in autoimmune rheumatic disease: potential clinical applications and perspectives

**DOI:** 10.1186/s41232-025-00369-2

**Published:** 2025-02-20

**Authors:** Atsuko Tsujii Miyamoto, Hiroshi Shimagami, Atsushi Kumanogoh, Masayuki Nishide

**Affiliations:** 1 Department of Respiratory Medicine and Clinical Immunology, Graduate School of Medicine, Osaka University, Suita, Osaka Japan; 2 Department of Immunopathology, World Premier International Research Center Initiative (WPI), Immunology Frontier Research Center (Ifrec), Osaka University, Suita, Osaka Japan; 3 Department of Advanced Clinical and Translational Immunology, Graduate School of Medicine, Osaka University, Suita, Osaka Japan; 4 Integrated Frontier Research for Medical Science Division, Institute for Open and Transdisciplinary Research Initiatives (OTRI), Osaka University, Suita, Osaka Japan; 5 Center for Infectious Diseases for Education and Research (Cider), Osaka University, Suita, Osaka Japan; 6 Osaka University, Suita, Osaka Japan; 7 Center for Advanced Modalities and DDS (Camad), Osaka University, Suita, Osaka Japan

**Keywords:** Spatial transcriptomics, Single-cell transcriptome analysis, Rheumatoid arthritis, Systemic lupus erythematosus

## Abstract

Spatial transcriptomics is a cutting-edge technology that analyzes gene expression at the cellular level within tissues while integrating spatial location information. This concept, which combines high-plex RNA sequencing with spatial data, emerged in the early 2010s. Spatial transcriptomics has rapidly expanded with the development of technologies such as in situ hybridization, in situ sequencing, in situ spatial barcoding, and microdissection-based methods. Each technique offers advanced mapping resolution and precise spatial assessments at the single-cell level. Over the past decade, the use of spatial transcriptomics on clinical samples has enabled researchers to identify gene expressions in specific diseased foci, significantly enhancing our understanding of cellular interactions and disease processes. In the field of rheumatology, the complex and elusive pathophysiology of diseases such as rheumatoid arthritis, systemic lupus erythematosus, and Sjögren’s syndrome remains a challenge for personalized treatment. Spatial transcriptomics provides insights into how different cell populations interact within disease foci, such as the synovial tissue, kidneys, and salivary glands. This review summarizes the development of spatial transcriptomics and current insights into the pathophysiology of autoimmune rheumatic diseases, focusing on immune cell distribution and cellular interactions within tissues. We also explore the potential of spatial transcriptomics from a clinical perspective and discuss the possibilities for translating this technology to the bedside.

## Background

Transcriptome analysis is a technology that comprehensively analyzes levels of RNA expression in cells. Since 2009, transcriptome analysis at the single-cell level, referred to as single-cell RNA sequencing (scRNA-seq), has emerged [1]. This technique has significantly improved the ability to identify cellular heterogeneity in specific cell subsets and complex tissues. scRNA-seq has revealed disease-specific cell types, leading to a great understanding of pathophysiology and the development of new treatments [2–7]. However, single-cell separation requires tissue dissociation to identify individual cells, resulting in a loss of spatial information. Information such as the location of specific cells and how these cells interact with other cells is of interest. In the early 2010s, several methods for high-plex RNA sequencing with spatial information emerged [8, 9]. In 2016, the concept of spatial transcriptomics was introduced as a technique that preserves spatial information with transcriptome analysis [10]. This technique has rapidly improved in terms of spatial resolution and high multiplexing capacity, with the development of techniques such as next-generation sequencing (NGS). To date, spatial transcriptomics has become an essential technique for elucidating the localization of disease-specific cell types in tissues [11].

In the field of rheumatology, single-cell transcriptome analysis has revealed that different cell types play important roles in the pathogenesis of disease [12]. For example, specific types of synovial fibroblasts (SFs) and macrophages play important roles in joint inflammation associated with rheumatoid arthritis (RA) [4, 13–17]. Spatial transcriptomics can further provide local information about these cells, revealing cell–cell interactions and where and how each cell functions in inflamed tissue. Thus, the combination of single-cell transcriptome analysis and spatial analysis leads to a deeper understanding of the dynamics of disease pathogenesis. In this review, we provide an overview of the development of spatial transcriptomics. In addition, we review current findings in autoimmune rheumatic disease on the basis of spatial transcriptomics and discuss how researchers have explored pathogenesis and novel therapeutic targets in rheumatology.

### Advances in spatial transcriptomics

**Fig. 1:**
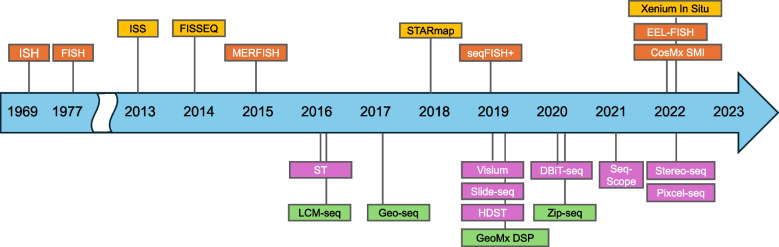
Timeline showing the development of spatial transcriptomics technologies. The historical progression of spatial transcriptomics technologies is shown on a timeline. Each color represents a distinct method: orange for in situ hybridization, yellow for in situ sequencing, purple for in situ spatial barcoding, and green for micro-dissection methods. ISH, in situ hybridization; FISH, fluorescence in situ hybridization; MERFISH, multiplexed error-robust fluorescence in situ hybridization; seqFISH, sequential fluorescent in situ hybridization; EEL-FISH, enhanced electric FISH; SMI, spatial molecular imager; ISS, in situ sequencing; FISSEQ, fluorescent in situ sequencing; STARmap, spatially resolved transcript amplicon readout mapping; ST, spatial transcriptomics; HDST, high definition spatial transcriptomics; DBiT-seq, deterministic barcoding in tissue for spatial omics sequencing; Stereo-seq, spatial enhanced resolution omics sequencing; Pixcel-seq, polony-indexed library sequencing; LCM, laser capture microscopy; DSP, digital spatial profiling

**Fig. 2:**
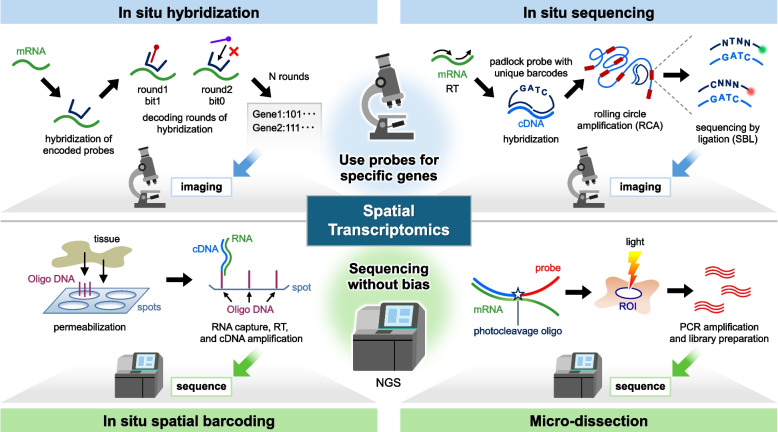
Mechanisms of representative spatial transcriptomics methods. Spatial transcriptomics methods are categorized into four representative methods. The two methods on the top row require the preparation of gene-specific probes. In situ hybridization directly analyzes the distribution of genes by hybridizing probes to mRNA in situ. In situ sequencing detects RNA by performing sequencing reactions within tissue samples by amplifying cDNA. The two methods shown on the bottom row employ unbiased sequencing techniques. In situ spatial barcoding uses oligo DNA probes to capture RNA at each spot in the tissue on a glass slide, followed by RT, cDNA amplification, and PCR. Microdissection-based methods analyze dissected ROIs within the tissue sample. cDNA, complementary DNA; RCA, rolling circle amplification; SBL, sequencing by ligation; RT, reverse transcription; NGS, next-generation sequencing; PCR, polymerase chain reaction; ROI, region of interest

The concept of detecting the expression of specific genes in tissues was demonstrated in 1969 with in situ hybridization [18]. Spatial transcriptomics has rapidly expanded with the development of various technologies (Fig. 1), currently classified into four major groups: in situ hybridization, in situ sequencing, in situ spatial barcoding, and microdissection-based methods [19–27] (Fig. 2). In situ hybridization and in situ sequencing methods have developed independently; however, the boundaries between them have become blurred as their protocols partially overlap.

#### In situ hybridization

In situ hybridization directly analyzes gene distribution by hybridizing probes to mRNA in situ (Fig. 2 upper-left). Historically, RNA was detected with radioactive materials to obtain location information [28]. Fluorescence in situ hybridization (FISH) uses complementary DNA (cDNA) probes with fluorescence [29], which has become an invaluable tool in clinical practice for the diagnosis of cancer and genetic disorders. However, overlapping spectra posed a problem for detecting a large number of transcripts simultaneously. Multiplexed error-robust fluorescence in situ hybridization (MERFISH) is a solution to this problem [8]. In this technique, an N-bit binary code word is assigned to each gene. Fluorescence-labeled readout probes hybridize to the corresponding sequences and a fluorescent signal is generated at the site of hybridization. After each round of hybridization, the readout probes are cleaved. The presence of a signal at a given site is assigned a “bit-1,” while the absence of a signal is assigned a “bit-0.” After N rounds of hybridization, the N-bit binary code is used to identify the specific mRNA species, allowing for the detection of approximately 10,000 mRNAs.

As a result of recent advances, sequential fluorescence in situ hybridization (seqFISH) + uses 20 pseudocolors for readout probes to improve the multiplexing resolution of the target site and reduce binary code detection error [30]. Enhanced electric FISH (EEL FISH) has enhanced the capacity of hybridization using electrophoresis. It also avoids non-specific binding to other cellular components [31]. CosMx Spatial Molecular Imager (CosMx SMI, NanoString) is a commercial high-plex in situ imaging chemistry platform that enables the detection of over 1000 RNAs at subcellular resolution [32]. The advantages of this method include its high detection power and multiplexing capacity at the single-cell resolution level. The disadvantages are the high cost per experiment, challenges in detecting short mRNAs, and the limitation that only predetermined targets can be detected.

#### In situ sequencing

In situ sequencing detects RNA by performing sequencing reactions within tissue samples (Fig. 2 upper-right). This method amplifies the target signal in situ using rolling circle amplification (RCA) and identifies base signals through microscopic examination [9]. This method begins with tissue fixation, followed by the conversion of mRNA into cDNA via reverse transcription (RT). The cDNA serves as the template for subsequent sequencing steps. Padlock probes, circularizable DNA molecules designed to target specific sequences, hybridize to the cDNA and are ligated at the target site. The circularized probes undergo RCA, producing DNA nanoballs. The sequence is decoded using a method called sequencing-by-ligation (SBL). SBL uses a primer to bind to the target sequence and ligates a fluorescence tag. In situ sequencing technology includes fluorescent in situ sequencing (FISSEQ) [33] and spatially resolved transcript amplicon readout mapping (STARmap) [34]. To obtain sufficient brightness for imaging, RNA must be converted to cDNA and amplified in the cell. Xenium in situ (10 × Genomics) is a commercial platform for in situ sequencing. It covers up to 5000 genes and allows for analysis at the single-cell level of resolution [35]. By amplifying RNAs by RCA and using in situ hybridization, it achieves high sensitivity. The advantages of this method include its high resolution at both the single-cell and single-molecule levels, as well as its applicability to thick tissue sections. The disadvantages include the difficulty of amplifying cDNA within the limited space inside the cell and the limitation that only predetermined targets can be detected.

#### In situ spatial barcoding (spatial capturing)

In situ spatial barcoding, also known as spatial capturing, uses oligo DNA probes to capture RNA specifically at barcoded spots in the tissue on a glass slide, which is then processed for RT and cDNA amplification (Fig. 2 lower-left). Transcriptome data is obtained separately from the imaging, which is a major difference between this method and in situ hybridization or in situ sequencing methods. The oligo DNA consists of an adapter, spatial barcode, unique molecular identifier (UMI), and poly T. Each spot has a unique spatial barcode sequence so that each gene can be mapped to a position after sequencing. The distance between spots defines spatial resolution. The UMI is a random sequence of several bases. When the same UMI is detected from a cDNA with the same sequence, it is identified as an overamplified cDNA. The number of copies of annealed mRNA is counted for quantification. In situ spatial barcoding was first demonstrated by Stahl et al. [10]. It is currently commercially available as Visium (10 × Genomics). In this platform, oligo DNA probes containing a barcode sequence are anchored to specific spots on a glass slide. The original Visium platform has a spatial resolution of 55 µm, which is insufficient for single-cell analysis. However, the recently developed Visium HD achieves a significantly higher spatial resolution (~ 2 µm), enabling single-cell and even subcellular levels of transcriptomic analysis. With other in situ spatial barcoding methods such as Slide-seq [36] and high-definition spatial transcriptomics (HDST) [37], microbeads with numerous oligo DNAs are scattered on the substrate and directly attached to the glass slide. Slide-seq employs a randomly distributed bead pattern, which requires computational reconstruction of spatial coordinates. HDST features a high-density, regularly spaced bead array, which enables more accurate spatial mapping. Slide-seq and HDST have improved the spatial resolution to 10 µm and 2 µm, respectively. However, the capture efficiency is not sufficient, and single-cell level analysis has not been achieved.

Microfluidics-based technologies have been introduced to further reduce channel size. Deterministic barcoding in tissue for spatial-omics sequencing (DBiT-seq) [38] is a technique that combines microfluidics and NGS. Polydimethylsiloxane microfluidic chips create unique barcodes with channel sizes of 10, 25, or 50 µm. Microfluidic systems allow for highly accurate control over small volumes of fluids, enabling precise manipulation of chemical and biological reactions. However, these systems do not reach the single-cell level due to limited channel capacity and the presence of empty spaces. In addition, the analysis size is limited to approximately 1mm^2^. Polony-indexed library sequencing (Pixel-seq) [39] and Seq-scope [40] are methods that use NGS to achieve single-cell level resolution. Tissue is placed on barcoded spots, permeabilized and mRNA is captured. After RT, the cDNA is submitted for NGS. Seq-scope has an ultra-high density of unique barcoded spots, which allows for higher spatial resolution. Spatial enhanced resolution omics sequencing (Stereo-seq) [41] uses DNA nanoballs as sequencing chips. DNA nanoballs are circular DNA templates generated by RCA, resulting in 400 spots per 100 μm ^2^ in area. After the tissue is permeabilized on the array, RNA is captured on the array and cDNA is collected for NGS.

Thus, spatial barcoding is a recent breakthrough in the field of spatial transcriptomics with improved capture efficiency of RNA transcription and detection depth per area; the number of UMIs per area is increasing, with approximately 5000 UMIs per 10 μm^2^ for DBiT-seq and 1000 UMIs per 10 μm^2^ for Stereo-seq, Seq-Scope, and Pixel-seq, respectively. In terms of tissue types, initially, only fresh frozen tissue was available for analysis, but currently, Visium and DBiT-seq can be used with formalin-fixed, paraffin-embedded (FFPE) tissue. Note that the current quality of RNA detection in FFPE tissue is lower than in fresh tissue. Further technological advances are expected as researchers have easier access to spatial analysis when semi-permanently stored FFPE tissue becomes widely available for experiments. The advantages of this method are the ability to perform transcriptome-wide analyses by capturing RNA, as well as providing single-cell level analysis on specific platforms. However, a disadvantage is the challenge of optimizing the balance between single-cell resolution and capture efficiency.

#### Microdissection-based methods

Microdissection-based methods analyze dissected regions of interest (ROIs) within a tissue sample (Fig. 2 lower-right). Laser capture microscopy (LCM) [42] was introduced in 1996. In this method, a narrow-diameter, high-energy laser can precisely cut out ROIs by burning off the contours of the target cell population. Recent technical advances in microarray and RNA-seq technologies have enabled LCM to be combined with Smart-seq2 (LCM-Seq　[43] and geographical position sequencing (Geo-Seq) [44]) to extract RNA from the targeted ROI more efficiently. However, the lack of spatial resolution below the laser diameter, which is typically a few micrometers (0.5 to 10 µm), is a limitation of the LCM method. Light-based detection methods extract transcriptomic information in the light-irradiated region and achieve finely shaped ROIs of several hundred nanometers. The ZipSeq [45] method uses oligo DNA conjugated to antibodies against cell surface antigens. A photocleavable blocker is attached to the oligo DNA and is designed to anneal to cells only when the blocker is cleaved by light. The cells in the ROI are fluorescently labeled by exposure to light. The tissue is then enzymatically dissociated and the fluorescent cells are separated using flow cytometry for subsequent scRNA-seq. GeoMx digital spatial profiling (DSP, NanoString) [46] uses oligo DNAs with an ultraviolet (UV) photocleavage linker and a barcode sequence as the mRNA antisense probe. The barcode is designed as a unique sequence for each gene. After hybridization to the tissue, UV light cleaves the linker, which can be collected and sequenced to obtain a gene expression profile in the ROI. Thus, light-based detection methods gather expression information in the ROI with high sensitivity. Spatial resolution reaches the single-cell level for ZipSeq and the diffraction limit (several hundred nanometers) for DSP. The advantage of this method is its resolution at the single-cell level or smaller and its ability to the codetection of proteins. A limitation is the labor-intensive manual selection of a limited number of ROIs, which hinders comprehensive whole-tissue transcriptomics analyses.

### Spatial transcriptomics in rheumatoid arthritis

**Fig. 3:**
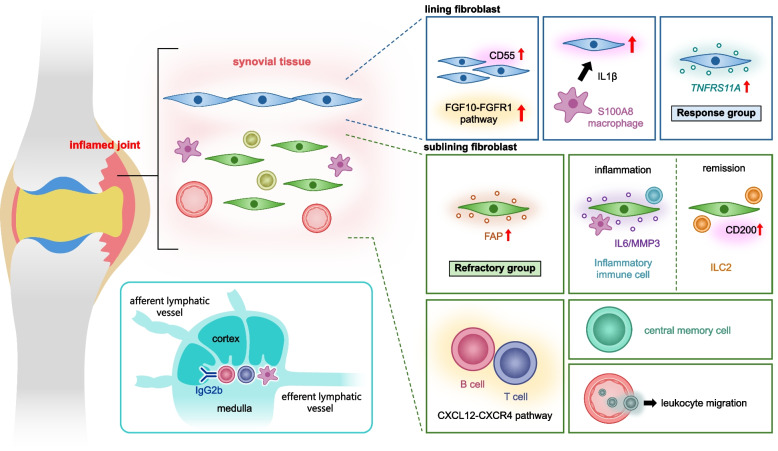
Application of spatial transcriptomics to rheumatoid arthritis. Key molecular pathways and cell interactions in RA synovium are highlighted. In the lining fibroblast region, the FGF10–FGFR1 signaling pathway is enhanced in CD55 + cells especially in refractory cases. Activation of these fibroblasts is driven by IL-1β from macrophages. TNFRS11A expression is upregulated in lining/ superficial sublining fibroblasts of treatment-responsive patients. Patients with refractory RA have increased FAP expression in the deep sublining fibroblast region. During inflammation, IL-6/MMP3 + fibroblasts are located near inflammatory immune cells. During remission, ILC2 cells are located near CD200 + fibroblasts. The CXCL12–CXCR4 pathway–induced interactions between B and T cells, the predominance of central memory cells, and pathways like leukocyte migration have been explored. In the lymph nodes, patients had reduced lymphatic flow and immunoglobulin switching, which are influenced by interactions between macrophages and T cells. RA, rheumatoid arthritis; FGF, fibroblast growth factor; IL, interleukin; FAP, fibroblast activation protein-α; MMP3, matrix metalloproteinase 3; ILC2, type 2 innate lymphoid cell

RA is a common autoimmune inflammatory disease characterized by inflammation of synovial tissue in the joints. RA affects approximately 0.5–1% of the population. Patients with RA suffer from disability in their daily lives due to joint inflammation and bone destruction [47]. To date, single-cell analyses of RA have revealed novel immune cell subtypes [4, 6, 48, 49] and synovial cell diversity [5, 17, 50–52]. Spatial analysis focusing on damaged joints has become increasingly important in elucidating local interactions between immune cells and synovial cells (Fig. 3, Table 1).

**Table 1:** Spatial transcriptomics in rheumatoid arthritis

Year	Specimen	Number of patients	Control	Platform	Key findings	Ref
2019	Synovium	3 ACPA +	3 PsA	In situ spatial barcoding [10]	Central memory T cells are enriched in inflamed synovial tissue of RA	53
2022	Synovium	3 ACPA + and 3 ACPA-	N/A	In situ spatial barcoding [10]	In ACPA + RA, leukocyte migration pathways are enriched in TLO	55
2022	Synovium	2 ACPA + and 2 ACPA-	N/A	Visium	CXCL12–CXCR4-mediated B cell T cell interactions play important roles in both ACPA- and ACPA + RA synovium	57
2022	Synovium	8 responders and 4 non-responders	N/A	GeoMx DSP	FAP expression is increased in the deep sublining region of non-responders	58
2023	Synovium	2	N/A	Visium	Fibroblasts responsive to IL-1β are localized in the lining region and contribute to synovial inflammation	61
2023	Lymph node (TNF-Tg mouse)	N/A	N/A	Visium	Loss of lymphatic flow through the affected joint-draining lymph nodes facilitates macrophages and T cell interactions, leading to IgG2 class switching	65
2024	Synovium	3	N/A	Visium	CD200⁺ fibroblasts are closely located with ILC2 in the synovium of remission cases	59
2024	Synovium	3 relapse and 3 remission cases	N/A	Visium	In refractory cases, CD55^+^ fibroblasts are abundant in the lining region and the FGF10-FGFR1 pathway is activated	62

*ACPA*, anti-cyclic citrullinated peptide antibody; *PsA*, psoriatic arthritis; *RA*, rheumatoid arthritis; *N/A*, not assessed; *TLO*, tertiary lymphoid organ; *DSP*, digital spatial profiling; *FAP*, fibroblast activation protein-α; *IL-1β*, interleukin 1 beta; *TNF*, tumor necrotizing factor; *ILC2*, type 2 innate lymphoid cell; *FGF*, fibroblast growth factor

In 2019, the first spatial transcriptomics in RA was conducted. It compared the transcriptome profiles of synovial tissues from patients with RA and psoriatic arthritis (PsA) [53]. Three patients with each disease were recruited and the in situ spatial barcoding [10] was used. In RA synovial tissues with immune cell infiltration, *CXCL9*, *LTB*, and *CD3E* were listed as the top differentially expressed genes (DEGs) in infiltrating cells. Pathway analysis showed that pathways involved in immune cell interactions were upregulated in these cells Furthermore, cell type assignment using the xCells [54] revealed that central memory T cells were enriched in RA synovial tissue whereas effector memory T cells were predominant in PsA tissue.

A study performed spatial transcriptomics in three-dimension (3D) synovial structures using in situ spatial barcoding by Stahl et al. [10]. Using synovial tissues from three anti-citrullinated protein antibodies (ACPA) + and three ACPA − RA patients, 3D structures were constructed [55]. Cluster analysis was performed to clarify characteristic signals and infiltrating cell types in each synovial region. Regions with high leukocyte infiltration were defined as tertiary lymphoid organ (TLO) regions [56]; *CD52* and *MS4A1* were characteristic genes in leukocytes that had infiltrated TLOs. In addition, genes related to TLO formation (e.g., *LTB*, *CCL19*, and *CCL21*), and genes essential for B cell–T cell crosstalk (e.g., *CXCL13*, *CD52*, and *MS4A1*), were upregulated in areas with infiltration in ACPA + patients. In ACPA − RA, *CD52* and *MS4A1* expression in the TLO was lower than in ACPA + RA. Pathway analysis showed that genes enriched in the TLO are mainly associated with signals such as leukocyte migration in ACPA + RA, while signals involved in extracellular matrix degradation and cell growth regulation were upregulated in ACPA − RA. Thus, spatial transcriptomics revealed that different cytokine pathways are enriched in specific disease foci leading to a better understanding of pathogenic TLO formation. In a study focusing on synovial tissue from two patients with ACPA + RA and two patients with ACPA − RA at the time of diagnosis, the combination of scRNA-seq and Visium revealed that B cells were located in the vicinity of T cells regardless of seropositivity status [57]. The CXCL12–CXCR4 axis is responsible for B cell maturation and survival. B cell receptor sequencing showed that memory B cells and plasma cells are clonally expanded in this niche. Plasma cells from ACPA + patients showed autoreactivity to citrullinated protein, suggesting local maturation of ACPA in synovial tissue. By integrating scRNA-seq and spatial transcriptomics, this study showed that the lymphoid cell niche, where T cells and B cells interact, is present even at the onset of ACPA + RA, which might help guide treatment selection in early-stage patients.

Pathogenic fibroblasts play an important role in the pathogenesis of RA. Single-cell analysis has revealed the diversity of fibroblasts; numbers of CD34^−^THY1^+^SFs are increased in RA. They are involved in bone destruction and inflammatory cytokine production [5, 17, 52]. To date, spatial transcriptomics has revealed the location of pathogenic fibroblasts and their interactions with surrounding immune cells, which is becoming increasingly important for a deeper understanding of pathology.

The R4RA study is a clinical trial designed to demonstrate histological differences in the efficacy of drugs such as rituximab (RTX) and tocilizumab (TCZ) in patients with RA [58]. In this study, spatial transcriptomics was performed using GeoMx DSP on RTX treatment responders (*n* = 4), TCZ treatment responders (*n* = 4), and treatment-resistant patients (*n* = 4). They divided the tissues into three ROIs: lining/superficial sublining, deep sublining, and lymphoid aggregates. Spatial transcriptomics was performed for each ROI. Gene expression profiles were significantly different between the treatment-responsive and treatment-refractory groups. The expression of fibroblast activation protein-α (FAP), a fibroblast marker, was increased in the deep sublining region of patients with refractory disease. *TNFRSF11A* was found in the lining/superficial sublining in the responder groups. *TNFRSF11A* encodes receptor activator of nuclear factor κB, which regulates osteoclast differentiation and activation. Thus, local transcriptomics associated with treatment response provides insights into the cause of poor response and additional therapeutic targets for poor responders. In mouse models of RA and PsA, single-cell analysis of SFs showed that the number of CD200^+^ fibroblasts increases in synovial tissue following treatment with interleukin (IL)−17 inhibitors [59]. These fibroblasts interact with type 2 innate lymphoid cells (ILC2s). In synovial tissues from three patients with RA, spatial transcriptomics showed that the number of CD200^+^DKK3⁺ fibroblasts increased and they co-localized with ILC2s after treatment, mainly in areas without apparent inflammation. These fibroblasts are significantly different from IL-6^+^ and matrix metalloproteinase-3 (MMP3)^+^ inflammation-related fibroblasts in inflamed tissue, suggesting the pathogenetic significance of dynamic changes in fibroblasts.

In a study focusing on the diversity of fibroblast-like synoviocytes (FLSs), four types of FLSs were detected using scRNA/assay for transposase-accessible chromatin　(ATAC)-seq [60]: activated lining fibroblasts, resting lining fibroblasts, activated sublining fibroblasts, and resting sublining fibroblasts [61]. The activated and resting states were classified on the basis of the presence of inflammatory responses and cytokine signaling. scATAC/RNA-seq of FLSs revealed that stimulation with a combination of tumor necrosis factor (TNF) and interferon (IFN)-γ drives the activated sublining FLS state, while the combination of TNF, IFN-γ, and IL-1β drives the activated lining FLS state. Visium analysis showed that each type of fibroblast is spatially distinct, with no distinct boundaries between the enriched regions of activated and resting fibroblasts. However, some specific types of fibroblasts could be distinguished. IL-1β–responsive fibroblasts were located in the lining compartment and co-localized with gene signatures such as S100A8^+^ tissue macrophages. These data suggest that IL-1β derived from macrophages and infiltrating activated monocytes is involved in FLS activation. A study using Visium on synovial tissue from six patients with RA focused on CD55^+^ fibroblasts. In patients with relapses, these fibroblasts proliferated in multiple layers, whereas in patients with remission, they were limited to a few superficial layers [62]. Cathepsin K (CTSK)^+^ macrophages were found in close proximity to CD55^+^ fibroblasts in relapse RA cases. Pathway analysis further showed that the human fibroblast growth factor (FGF)10–FGFR1 pathway is enhanced in CD55^+^ fibroblasts in patients with refractory disease. This pathway was related to increased inflammation and joint damage. In experiments with pannus cell culture, recombinant FGF10 promoted bone erosion, while blockage of the FGFR1 signaling pathway has the opposite effect, suggesting that inhibition of the FGF10–FGFR1 axis has therapeutic potential.

It is well known that lack of lymphatic drainage and altered B cell localization are important in the pathogenesis of RA [63, 64]. By Visium analysis of lymph nodes in a mouse model of synovitis, TNF-Tg mice have a macrophage receptor with collagenous structure (MARCO)^+^ subtype of lymphatic endothelial cells that were predominantly located in the lymph sinuses [65]. In the advanced synovitis group, IgG2b^+^ plasma cells were present around the MACRO^+^ parafollicular medulla and the number of CD6^+^ T cells in this region was higher than in wild-type mice. Cell–cell interaction analysis showed that activated leukocyte cell adhesion molecule (ALCAM) on macrophages and CD6 on T cells are involved in T cell activation, contributing to IgG2b class switching via the T cell co-stimulatory pathway. This study suggests that loss of lymphatic flow through lymph nodes that drain the affected joint might facilitate interactions between macrophages and T cells, leading to IgG2 class switching.

### Spatial transcriptomics in systemic lupus erythematosus

Systemic lupus erythematosus (SLE) is an autoimmune disease with a wide range of clinical manifestations, including the kidney, blood cell, and skin involvement [66]. Type I interferon signaling plays an essential role in the pathogenesis and upregulation of interferon-stimulated genes (ISGs) are widely detected in tissue cells and blood cells. Therefore, the inhibition of interferon signaling offers therapeutic benefits [67, 68] (Table 2).

**Table 2:** Spatial transcriptomic analysis in other rheumatic diseases

Year	Specimen	Number of patients	Control	Platform	Key findings	Ref
Systemic lupus erythematosus						
2022	Kidney (MRL-*Lpr* mouse)	N/A	N/A	Visium	Two types of macrophages, TrMacs and MoMacs are identified in glomeruli	72
2023	Kidney	2 lupus nephritis	N/A	Visium	APOE^+^ monocytes are increased around glomeruli	69
2023	Hippocampus and hindbrain (*Sle1*, *Yaa* mouse)	N/A	N/A	MERFISH	Type 1 IFN signatures are enhanced in the hindbrain and hippocampus of the NPSLE mouse model	73
2024	Kidney	4 lupus nephritis	4	GeoMx DSP	PI3Kα overactivation drives podocyte injury in glomeruli	70
2024	Kidney (mouse treated with TLR7 agonist)	N/A	N/A	Stereo-seq, Xenium In Situ	T cells and macrophages are closely located to podocytes	71
Sjögren’s syndrome						
2022	Salivary gland (*NOD.H-2b* mouse)	N/A	N/A	Visium	TYROBP Causal Network is enhanced in salivary glands of the SS model	75
Systemic scleroderma						
2023	Skin (bleomycin-induced model mouse)	N/A	N/A	Visium	Celastrol (YAP inhibitor) inhibits the fibrotic impact of bleomycin by inhibiting matricellular and inflammation pathways in fibroblast clusters	77
Idiopathic inflammatory myopathies						
2024	Muscle	3 IBM	2 IMNM, 3 controls	Visium	Cell stress and denervation pathways lead to type 2 fiber vulnerability in the IBM muscle	80
2024	Muscle (*Icos*^−/−^ NOD mouse)	N/A	N/A	GeoMx DSP	IFN-γ plays pathogenic roles in mitochondrial dysfunction of IIM muscle	81
Vasculitis						
2023	Temporal artery	9 GCA	7	GeoMx DSP	Macrophage activation pathways are enhanced in the intima, media, and adventitia of temporal arteries in GCA	83
2024	Kidney	28 ANCA-GN	N/A	Visium	Th1 and Th17 cells are prominent in inflamed glomeruli and tubulointerstitial regions of ANCA-GN	85
IgG4-related disease						
2024	Submandibular gland	2 IgG4RD	N/A	Visium	Genes crucial in cell cycling and B cell differentiation/activation are upregulated in germinal centers of the IgG4RD salivary gland	87

*SLE*, systemic lupus erythematosus; *N/A*, not assessed; *TrMacs*, tissue-resident macrophages; *MoMacs*, monocyte-derived macrophages; *APOE*, apolipoprotein E; *DSP*, digital spatial profiling; *PI3Kα*, phosphoinositide 3-kinase alpha; *MERFISH*, multiplexed error-robust fluorescence in situ hybridization; *IFN*, interferon; *NPSLE*, neuropsychiatric systemic lupus erythematosus; *TLR*, Toll-like receptor; *Stereo-seq*, spatial enhanced resolution omics sequencing; *SS*, Sjögren’s syndrome; *SSc*, systemic scleroderma; *YAP*, yes-activated protein; *IBM*, inclusion body myositis; *IMNM*, immune-mediated necrotizing myopathy; *Icos*, inducible T cell co-stimulator; *NOD*, non-obese diabetic; *GCA*, giant cell arteritis; *ANCA-GN*, antineutrophil cytoplasmic antibody-associated glomerulonephritis; *IgG4RD*, IgG4-related disease

A study combining scRNA-seq and spatial transcriptomics of lupus nephritis (LN) detected apolipoprotein E (APOE)^+^ monocytes as a specific monocyte population in LN kidney tissue [69]. As APOE expression increased in monocytes and macrophages, antigen-presenting and interferon-producing capacity decreased accordingly. Spatial transcriptomics of two LN kidney tissue samples using Visium showed that APOE^+^ monocytes accumulate around the glomeruli. In addition, APOE^+^ monocytes were likely to migrate through newly formed lymphatic vessels in LN kidney tissue. This lymphangiogenesis plays a crucial role in transporting immune cells to inflamed areas and contributes to the progression of local inflammation. GeoMx DSP analysis of tissues from four patients with LN and four healthy controls highlighted that the phosphoinositide 3-kinases (PI3K)α pathways influence podocyte function and immune responses in LN kidney tissue [70]. Spatial transcriptomic analysis revealed increased expression of inflammation-related genes and podocyte dedifferentiation markers (e.g., *WNT4*) in LN glomeruli. Significant upregulation of pathways associated with podocyte damage such as WNT was observed in areas with high p-AKT activity. This study suggested that the PI3K-AKT pathway might be a potential therapeutic target and demonstrates that alpelisib, a selective inhibitor of the PI3Kα pathway, preserves podocyte homeostasis and reduces immune-mediated kidney inflammation in LN mouse models. A study used novel computational methods to analyze spatial transcriptomics data with subcellular resolution in a Toll-like receptor (TLR)7 agonist-induced LN mouse model [71]. The platforms used for spatial transcriptomics were Xenium In Situ and Stereo-seq, which allowed analysis at subcellular resolution. TopACT is a method that facilitates accurate cell annotation through mathematical algorithms. The combination of TopACT and scRNA-seq data enables more accurate cell annotation. In this study, the authors also applied TopACT to Xenium In Situ on human IgA nephropathy samples, demonstrating its applicability beyond the mouse model. In addition, they applied multiparameter persistent homology (MPH) to TopACT, enabling systematic and quantitative characterization of immune cell spatial patterns. The study revealed a unique peripheral ring-like distribution of immune cells around the glomeruli, particularly T cells and macrophages in the LN mouse model. Immune cells were located in close proximity to podocytes, suggesting their infiltration into the glomerular tissue rather than confinement to the vasculature. This observation provides new insights into the mechanisms of inflammation in LN and highlights how immune cell infiltration contributes to renal pathology. Notably, the study revealed a unique peripheral ring-like distribution of immune cells around glomeruli, particularly T cells and macrophages. In the MRL-*Lpr* mouse model of LN, two major renal macrophage populations were identified using the Visium platform and scRNA-seq: F4/80^hi^ TrMacs (tissue-resident macrophages) and F4/80^low^CD11b^hi^ MoMacs (monocyte-derived macrophages) [72] TrMacs were enriched around the glomeruli and expressed high levels of *Ccl8* to attract MoMacs to the vicinity. In addition, TrMacs can produce B cell tissue niche factors, suggesting that they play a role in supporting autoantibody-producing lymphoid aggregates. On the other hand, MoMacs were distributed in the cortical regions and had high expression of *Fcgr4* for FcγR-mediated immune complex responses. From a clinical point of view, the inhibition of TrMacs function might help reduce the recruitment of inflammatory monocytes into the kidney. Finally, this study further showed extensive similarities were observed with human kidney macrophages, too.

In a study on neuropsychiatric SLE (NPSLE), the *Sel1* mouse and the *Yaa* mouse were used for MERFISH on brain tissue [73]. In these animal models of NPSLE, the brain parenchyma of mice with symptoms of anxiety and fatigue had a significantly enhanced interferon pathway. The expression of ISGs was also increased in the hindbrain and hippocampus. Conversely, pathways related to cellular interactions and nervous system development were suppressed in astrocytes and oligodendrocytes. Type 1 interferon signaling was characteristic of various areas within the brain parenchyma, suggesting that IFNs may affect the behavior of mice with NPSLE by suppressing local cell–cell interactions.

### Spatial transcriptomics in other rheumatic diseases

#### Sjogren’s syndrome

Sjogren’s syndrome (SS) is an autoimmune disease in which lymphocytic infiltration affects exocrine glands [74]. Spatial transcriptomics was performed using Visium on salivary glands of NOD.B10Sn-H2b/J (*NOD.H-2b*) mice, a mouse model of SS [75]. The TYROBP casual network, a pathway that includes macrophage and myeloid cell activation markers, was upregulated in the salivary gland, especially within and around infiltration foci. In addition, the pathway associated with lipid metabolism was decreased in lacrimal gland epithelial cells. This metabolic shift might be a response to ongoing inflammation. These findings suggest that the macrophage-enriched metabolic network plays a central role in the inflammatory milieu of SS.

#### Systemic scleroderma

Systemic scleroderma (SSc) is an autoimmune disease characterized by tissue fibrosis [76]. The disease pathogenesis is driven by abnormal immune responses, microvascular inflammation, and excessive collagen production. While thickening and hardening of the skin are typical manifestations, internal organs such as the lungs, heart, kidneys, and gastrointestinal tract are also frequently affected.

A study using Visium demonstrated that celastrol, a yes-activated protein (YAP) inhibitor, alleviates fibrosis in SSc-like bleomycin-induced skin in mice [77]. Based on specific markers in healthy mouse skin tissue, four distinct cell types were identified: epithelial cells, papillary fibroblasts, reticular fibroblasts, and universal fibroblasts. Bleomycin treatment markedly reduced the expression of characteristic genes in the papillary and universal fibroblast clusters, while increasing gene expression in the reticular fibroblast and epithelial cell clusters. Celastrol mitigated the fibrotic effects of bleomycin by preventing these gene alterations. In particular, celastrol reduced the expression of genes involved in matricellular pathways (e.g., *Ccn2* and *Ccn1*) and matrix regulation (e.g., *Adam10* and *Col4A1*). Celastrol-sensitive genes were primarily associated with the Hippo (including YAP), mTOR, Wnt, and focal adhesion signaling pathways. This study highlights the potential of celastrol as an effective therapeutic agent for skin fibrosis by inhibiting fibrogenic gene expression programs.

#### Idiopathic inflammatory myopathy

Idiopathic inflammatory myopathies (IIMs) are a heterogeneous group of autoimmune disorders primarily characterized by chronic inflammation of the skeletal muscle, resulting in progressive muscle weakness. There are several subtypes of this disease, including anti-synthetase myositis, dermatomyositis, immune-mediated necrotizing myopathy, and inclusion body myositis (IBM) [78]. IBM is a slowly progressive myopathy characterized by the infiltration of cytotoxic T cells into muscle tissue, typically presenting with muscle weakness in the finger flexors and quadriceps [79]. Spatial transcriptomics using Visium has revealed a significant reduction in type 2 myonuclei, which are commonly associated with fast-twitch muscle fibers, in IBM muscle tissue compared with non-inflammatory controls or other myositis subtypes [80]. Furthermore, IBM muscle tissue has higher levels of cytotoxic T cells and conventional type 1 dendritic cells. Markers of cellular stress (e.g., *GADD45A* and *NORAD*) were highly expressed in myofibers, particularly in regions with severe inflammation. Elevated levels of *ACHE*, the gene encoding acetylcholinesterase, were also observed in association with *NORAD* expression, suggesting a potential mechanism for functional denervation at the neuromuscular junction that can further exacerbate muscle fiber degeneration. These findings suggest that the pathophysiology of IBM is associated with genomic stress, denervation, and inflammation, specifically impacting type 2A muscle fibers. In inducible T cell co-stimulator (*Icos)*^−/−^ non-obese diabetic (NOD) mice, which develop spontaneous muscle inflammation mimicking human IIMs, GeoMx DSP was employed to analyze muscle tissue from four *Icos*^+/+^ mice and four *Icos*^−/−^ mice [81]. Myofibers were classified according to their proximity to immune cell infiltrates: PROX fibers (myofibers in close proximity, but not directly adjacent, to immune cell clusters), ADJ fibers (myofibers directly adjacent to immune infiltrates), and control fibers (myofibers from unaffected *Icos*^+/+^ NOD mice). PROX and ADJ fibers showed a marked reduction in the expression of mitochondrial genes (e.g., *Cox6a2* and *Ndufa4*) genes relative to control myofibers, with the reduction more evident in ADJ fibers. These data suggest that immune cell infiltration exacerbates mitochondrial dysfunction in nearby myofibers. In addition, RNA sequencing from human samples showed infiltrated regions with the lowest mitochondrial gene expression had higher expression of IFN-γ related genes (e.g., *GBP2* and *IFI30*), implicating IFN-γ as a key driver of mitochondrial dysfunction in these areas. Treatment with anti-IFN-γ antibodies was associated with reduced reactive oxygen species (ROS) production, improved mitochondrial ultrastructure, and restoration of respiratory enzyme activities (COX, NADH-TR, and SDH) in the muscle tissue, suggesting the therapeutic potential of targeting IFN-γ mediated pathways to reduce mitochondrial dysfunction in IIMs.

#### Giant cell arteritis

Giant cell arteritis (GCA) is a large-vessel vasculitis in adults, mainly affecting those over 50 years of age, with a particular predilection for the aorta and medium-sized arteries [82]. GeoMx DSP was performed on temporal artery tissue biopsied from nine patients with GCA and seven controls. For spatial transcriptomics, each anatomical region of the vessel was defined: intima, media, adventitia, and others [83]. Throughout the regions, genes associated with antigen presentation (*CD74*), macrophage activation (*CD68*), and vascular remodeling (*MMP* and *COL1A2*) were upregulated. These changes in gene expression were particularly evident in the intima, whereas the adventitia had fewer gene alterations. Pharmacogenomic network analysis integrating DEGs from each arterial layer suggested that *MMP*s and *CD74* might be therapeutic targets in GCA.

#### ANCA-associated vasculitis

Antineutrophil cytoplasmic antibody (ANCA)-associated vasculitis is a group of autoimmune diseases characterized by inflammation of small to medium blood vessels. It commonly affects the kidneys, lungs, and skin [84]. Single-cell transcriptome analysis and spatial transcriptomics using Visium on kidney biopsies from 34 patients with ANCA-associated glomerulonephritis (ANCA-GN) revealed distinct inflammatory niches within the kidney, particularly in inflamed glomeruli and tubulointerstitial regions [85]. T cell activation was identified as a key driver of inflammation in these regions with a prominent role for Th1 and Th17 cells, which produce pro-inflammatory cytokines such as IL-12 and IL-23. A digital pharmacology approach based on these transcriptomic profiles identified ustekinumab, a monoclonal antibody that targets IL-12 and IL-23, as a potential therapeutic agent. Treatment of patients who have refractory ANCA-GN with ustekinumab led to improvements in kidney function and a significant reduction of serum creatinine levels in all four patients.

#### IgG4-related disease

IgG4-related disease (IgG4RD) is characterized by tissue infiltration with IgG4-expressing plasma cells, which leads to fibrosis and the formation of mass-like lesions in various organs, including the pancreas, salivary glands, lacrimal glands, and kidneys [86]. One study combined bulk RNA sequencing and spatial transcriptomics of submandibular gland biopsy samples from patients with IgG4RD [87]. Spatial transcriptomics using Visium from two patients revealed high expression of genes related to cell cycle (e.g., *CDK1*) and B cell differentiation (e.g., *CD22* and *SPIB*), particularly within germinal centers. Clinically, the identification of *CDK1* as a key gene suggests that targeting this protein could offer a novel therapeutic approach for IgG4RD.

## Conclusions

Spatial transcriptomics has evolved significantly since its introduction in 2013. It now offers great promise for elucidating pathogenic mechanisms and identifying therapeutic targets in a variety of diseases. Initially applied in oncology [35, 88], this technology has deepened our understanding of the cellular microenvironment through insights into the interactions of immune and non-immune cells and pathways activated within specific regions of diseased organs. As discussed in this review, the use of patient-derived samples of inflamed tissues in rheumatic disease, including synovial tissues in RA, renal tissues in SLE, and muscle tissues in inflammatory myopathies, has expanded.

Despite its potential, the clinical translation of spatial transcriptomics faces challenges such as high costs and complex experimental methodologies. Moreover, the majority of the current evidence in rheumatology is derived from small-scale studies, often involving fewer than 10 patients. This limitation makes it difficult to generalize the results to a larger patient population. To overcome these barriers, recent advancements in commercially available platforms such as GeoMx DSP, Visium, Xenium In Situ, and CosMx SMI are improving accessibility and capability, thus contributing to the rapid expansion of this field. As spatial transcriptomics continues to advance, it will increasingly become a powerful tool in the diagnosis, classification, and treatment of complex autoimmune diseases. Combining detailed spatial data with practical clinical observations offers a promising pathway to advanced disease management and precision medicine.

## Data Availability

Not applicable.

## Abbreviations

scRNA-seq: Single-cell RNA sequencing
NGS: Next-generation sequencing
SF: Synovial fibroblast
RA: Rheumatoid arthritis
FISH: Fluorescence in situ hybridization
cDNA: Complementary DNA
MERFISH: Multiplexed error-robust fluorescence in situ hybridization
seqFISH: Sequential fluorescence in situ hybridization
EEL FISH: Enhanced electric FISH
SMI: Spatial molecular imager
RCA: Rolling circle amplification
RT: Reverse transcription
SBL: Sequencing by ligation
FISSEQ: Fluorescent in situ sequencing
STARmap: Spatially resolved transcript amplicon readout mapping
UMI: Unique molecular identifier
HDST: High-definition spatial transcriptomics
DBiT-seq: Deterministic barcoding in tissue for spatial-omics sequencing
Pixcel-seq: Polony-indexed library sequencing
Stereo-seq: Spatial enhanced resolution omics sequencing
FFPE: Formalin-fixed, paraffin-embedded
ROI: Region of interest
LCM: Laser capture microscopy
DSP: Digital spatial profiling
UV: Ultraviolet
PsA: Psoriatic arthritis
DEG: Differentially expressed genes
3D: Three dimension
ACPA: Anti-cyclic citrullinated peptide antibody
TLO: Tertiary lymphoid organ
RTX: Rituximab
TCZ: Tocilizumab
FAP: Fibroblast activation protein-α
IL: Interleukin
ILC2: Type 2 innate lymphoid cell
MMP3: Matrix metalloproteinase 3
FLS: Fibroblast-like synoviocytes
ATAC: Assay for transposase-accessible chromatin
TNF: Tumor necrosis factor
IFN: Interferon
CTSK: Cathepsin K
FGF: Fibroblast growth factor
MARCO: Macrophage receptor with collagenous structure
ALCAM: Activated leukocyte cell adhesion molecule
SLE: Systemic lupus erythematosus
ISG: Interferon-stimulated genes
LN: Lupus nephritis
APOE: Apolipoprotein E
PI3K: Phosphoinositide 3-kinases
TLR: Toll-like receptor
TrMacs: Tissue-resident macrophages
MoMacs: Monocyte-derived macrophages
NPSLE: Neuropsychiatric SLE
SS: Sjogren’s syndrome
SSc: Systemic scleroderma
YAP: Yes-activated protein
IIM: Idiopathic inflammatory myopathies
IBM: Inclusion body myositis
Icos: Inducible T cell co-stimulator
NOD: Non-obese diabetic
ROS: Reactive oxygen species
GCA: Giant cell arteritis
ANCA: Antineutrophil cytoplasmic antibody
GN: Glomerulonephritis
IgG4RD: IgG4-related disease
